# Rethinking health policy student practicums through the application of the multiple streams framework: A case study

**DOI:** 10.1177/08404704211009231

**Published:** 2021-05-12

**Authors:** Kelly Anderson, Michael Heenan

**Affiliations:** 1 7938 University of Toronto, Toronto, Ontario, Canada.; 2 3710 McMaster University, Hamilton, Ontario, Canada.

## Abstract

Many university and college programs offer co-op placements or practicums as part of their curriculum, with the aim of providing real-world experience and opportunity for students to apply theory to practice. These practicums are not always grounded in the underlying management or policy theories the program teaches, instead they often focus on universal attributes such as task performance or general leadership. This case study describes how a University of Toronto Health Policy and Management student and an Executive from Ontario’s Ministry of Health redesigned the student’s practicum to be grounded in Kingdon’s Multiple Stream Policy Framework. The case demonstrates how the theoretical framework was applied to enhance their weekly mentorship discussions, and organize the student’s learnings relating to the Ministry’s policy on hospital capacity during the COVID-19 pandemic by viewing the work through the framework’s five streams.

## Introduction

The academic world regularly deploys field practicums as a part of learning curriculums. They exist in many institution’s curriculums as an opportunity for students to gain real-world experience. Universities offer a wide variety of these internship programs, often varying in title and composition. These programs have been described as experiential learning, placements, internships, co-ops, field studies, administrative residencies, and the list goes on.^1–3^ In addition to the diversity in terminology, these practicums also differ in duration, compensation, academic deliverables, and overall learning objectives.^1,3,4^

At the University of Toronto, the objective of the Institute of Health Policy, Management and Evaluation (IHPME) Master of Health Science (MHSc) practicum is to “broaden the students’ appreciation for and skills in managing health service organizations by allowing them to evaluate, test, and further develop their leadership competencies in a practical setting.”^5^ This 8- or 12-week practicum aims to offer flexibility for master’s students to pursue their practicum with an organization which aligns with their learning goals, given their past work experience, area of interest, and career objectives. Specific deliverables for this practicum include a learning contract, reflective journaling, and performance evaluations. The learning contract is co-developed by the student and the Executive to facilitate the identification of goals that meet the needs of both the student and their practicum organization. As is the case for the IHPME MHSc curriculum, the learning goals for this practicum are tied to the National Centre for Healthcare Leadership Competency Model, aimed to support well-rounded leadership competency growth.^5^

This case study describes the method a University of Toronto MHSc (Health Administration) student and an Executive from Ontario’s Ministry of Health used to ground the student’s Ministry practicum in the theoretical constructs of the Multiple Stream Framework (MSF). The student and Executive used the framework to enhance their weekly mentorship meetings which included targeted reflection and discussion of the application of the framework’s five streams. In addition, the case demonstrates how the application of the MSF during the student’s practicum amplified the student’s participation in and learning about the development of the Ministry’s policy on hospital capacity during the COVID-19 pandemic.

## Narrative review: Benefits and challenges of student practicums

The problem of inert knowledge, failure to apply concepts learned in the classroom to real-world problems, is widely recognized.^2,6,7^ Exposure to complex problems during practicums increases a student’s awareness and ability to apply theory to practice.^8^ These opportunities enable the student to assess the value, relevance, and integration of multiple perspectives to decision-making processes. Learning to see beyond a black and white solutions approach, appreciating “grey areas” and understanding there may not be one right answer, is fundamental knowledge for students to acquire.^3^

During practicums, students can optimize their learnings by engaging in formal mentorship discussions with the Executive. These discussions provide a platform for intentional reflection and opportunity to discuss complex problems that the student has observed. Such mentorship discussions also offer the student insight into how a leader or organization might identify potential barriers and develop solutions to problems. These conversations facilitate the transition of inert knowledge to application in real-world contexts, a process that is optimized through the Executive’s guidance in making the concepts explicit.^4^ The key is to turn the practicum into a cognitive apprenticeship in which reflection is intentionally integrated, and problem finding is afforded the same weight as problem solving.^2^ Research across fields has demonstrated that this type of contextually relevant, real-world learning opportunity, which fosters reflective observation and debriefing, aligns with core adult learning principles, offering the platform to maximize learning from such practicums.^9–11^

Despite the aim of post-secondary and graduate-level practicums to facilitate knowledge application in real-world settings, their benefits are not always realized to their full potential.^2^ Leveraging a framework to act as guideposts for learnings during the practicum helps to organize the knowledge, skill, and abilities acquired from this learning experience.^8,12^ Additionally, mapping learnings to a framework enables the student to monitor advancements in their knowledge and skills, and offers a common language to facilitate discussion of these learnings between student and Executive.^12^ Furthermore, while there is often clarity in a single practicum learning experience, there often lacks cohesion across multiple learning experiences.^12^ Tying all learnings back to a foundational framework highlights the integration of these experiences, which in turn maximizes learnings garnered by the student.^12^

## Multiple streams framework

The MSF is a theoretical construct that describes how policy moves from the idea stage to implementation through the convergence of five independent streams.^13,14^ The five streams can be summarized as follows: (1) problem stream: where a problem is identified and defined, (2) policy stream: where ideas are created to address the problem, (3) political stream: where stakeholder interests and institutions impacted by problem and policy alternatives are identified, (4) policy window: the timing opportunity when the above streams unite to ensure there is widespread attention to the issue, and a willingness to adopt change, (5) policy entrepreneurs: the key actors who advocate for the policy alternatives and problem description.^13,14^ In order for the policy idea to reach the agenda setting and adoption stages of the policy lifecycle, it is believed these streams must unite through the policy window with the support of a policy entrepreneur who frames problems to indicate change is needed.^14^ A successful policy is also positioned within the government’s political agenda and has broad stakeholder support.^14,15^

## Structured mentorship via MSF

Given the past challenges practicums have had in ensuring learnings for the student are relevant, as noted by the narrative review, the student and Executive agreed to redesign the practicum to include a structured approach whereby the student’s learnings would be grounded in a theoretical framework. The MSF was chosen as the theoretical framework to structure the student’s practicum learnings given its five components could easily be adopted into a standard agenda for mentorship. This was particularly helpful as the practicum occurred during a time of rapid policy-making when the Ministry was responding to the COVID-19 pandemic.

Each week the student and Executive met to review the student’s deliverables and to reflect on the student’s practicum experiences. In each meeting the framework was addressed through a series of questions aimed at having the student identify where they witnessed the five streams of the framework in action. These questions included:

- Problem stream; what problems did you see the Ministry presented with this week?
- Policy stream; what ideas were generated in the Ministry to potentially address the problem? If none was created, why do you think there was a delay?
- Political stream; who do you think is impacted by the policy problem and who might we need to involve to address it?
- Policy window; do you think the problem can be resolved in a short, medium, or long-term window? Why?
- Policy entrepreneur; who was presenting the problems and ideas? What do you think his/her motivations are?

In addition to these questions, the student and Executive often discussed how the framework was applied to a current policy discussion and the potential challenges and barriers associated with the framework’s application.

## Using the multiple stream framework: A policy base example

The most significant policy the student and Executive worked on was the development of a new hospital capacity plan designed to address a shortage of hospital beds in anticipation of a second wave of COVID-19. The policy, which eventually added up to 3,141 acute, post-acute, and critical care beds in Ontario, was endorsed and funded by the Ontario government in the fall of 2020.^16^ Table 1 offers a summary of how the student and Executive aligned the COVID-19 hospital bedded capacity policy to the five streams of the MSF, while illustrating the lessons learned by the student.

**Table 1.** Viewing the COVID-19 hospital bedded capacity policy through the multiple stream framework; illustration of a master’s student’s practicum learnings

MSF component	Capacity policy inference	Student learnings
Problem stream: Where a problem is identified and defined.	Provincial capacity data indicated an emerging capacity challenge in acute care during the COVID-19 pandemic. With a desire to ensure adequate capacity in the province, the Ministry examined the added capacity needed to care for patients with COVID-19 while not reducing surgical activity.	The student understood the size and significance of the capacity need across the system a result of different but intertwined data points such as increasing acute volumes, alternative level of care (ALC) rates, lack of long-term care (LTC) beds, and rising public health risks.
Policy stream: Where ideas are created to address the problem.	The Ministry developed a multi-pronged Fall preparedness plan of which hospital capacity was one pillar. The idea was to add hospital beds to not only aid in treating COVID-19 positive patients but aid the hospitals in addressing rising ALC rates due to a loss of LTC beds in the system (as a result of IPAC cohorting restrictions), increased admissions through ER (for those who delayed seeking care), and to address the surgical backlog (as a result of elective surgery postponement). The idea resulted in a hospital bedded capacity framework that opened over 3,000 new beds based on the criteria above. This policy was grounded in a tiered investment framework that established the type of bed needed, the location of that bed, and how long that bed should be opened for.	The student gained insight into how a specific hospital policy option was created and framed within a larger plan. The student completed a pan-Canadian jurisdictional scan regarding how bedded capacity should be added, enabling the student to gain insight into how other innovative approaches support the creation of a local solution to a common problem. The student saw how the development and use of a tiered investment framework synthesized a complex problem and positioned the policy options within the context of the government’s overall goals and philosophy for streamlined decision-making.
Political stream: Where stakeholder interests and institutions impacted by problem and policy alternatives are identified.	The Ministry proactively collaborated with its main delivery agency Ontario Health, Critical Care Services Ontario, the Ontario Hospital Association, and individual hospitals to identify capacity needs based on regional COVID prevalence, past utilization, and pre-pandemic hallway bed count.	The student observed the working relationships between significant stakeholders, how their individual interests informed the final policy construct, and how the collaboration ensured their ongoing support for the policy.
Policy window: The timing opportunity when the above streams unite to ensure there is widespread attention to the issue, and a willingness to adopt change.	The emergency nature of the pandemic created an environment where decision-makers not only wanted options to consider but required these solutions in a timely manner. Policy risks were assessed but in a much quicker manner than normal given the nature of the pandemic. The governments ask for a comprehensive Fall preparedness plan also created a window of alignment for hospitals so the new bedded capacity was seen as part of a broader response than a single stakeholder issue.	The student observed the pressure all stakeholders placed on the Ministry to make expedited policy decisions in a crisis environment. In addition, the student observed how policy can be made quickly when all parties recognize its urgency, as demonstrated by the COVID-19 response.
Policy entrepreneur: Key actor who pushes the policy alternatives and problem description.	Given the public health challenge of COVID-19, the policy entrepreneurs of this policy included all four of the province’s largest health stakeholders: The Ministry’s Hospital and Capital division who shared provincial capacity forecasting early and struck a call for hospitals to identify options. Ontario Health who manage local planning and delivery models and focused on ensuring regional balance in bed distribution was achieved. Critical Care Services Ontario who identified the number of Intensive Care beds needed. Ontario hospitals who provided input into local challenges and offer insights into how different bed types could be used to add capacity at their individual sites.	The student observed how the development and use of a tiered investment framework by the Ministry’s Hospital and Capital division synthesized a complex problem and positioned the policy options within the context of the government’s overall goals and philosophy for streamlined decision-making.

Abbreviations: ALC, alternative level of care; ER, Emergency Room; IPAC, Infection Prevention and Control; LTC, long-term care; MSF, multiple stream framework.

## Discussion

In considering this practicum within the context of this article’s narrative review that noted challenges related to a lack of structure and relevance to student learning goals, recommendations for schools facilitating experiential learning opportunities and host organizations arise. Structured weekly meetings between student and Executive offer space for the mentoring relationship to grow, and opportunity for the student to intentionally reflect on their learnings, enhanced through this shared dialogue. Adoption of a relevant framework helps to organize these reflective conversations, providing a mechanism to analyze the knowledge, skill, and abilities the student acquires from their practicum experience. In this case study, the MSF offered the cognitive infrastructure for the student to map their learnings against further deepened by the mentor encouraging the student to link and articulate their learnings against the five streams within the framework.

The Ministry’s policy on adding hospital capacity was highly complex, given the speed, cost, and stakeholder management impact during a pandemic. Applying the MSF to the practicum enabled the student to appreciate a holistic view of this complex problem and place links between each situational learning to the broader context. As an example of how this theoretical framework was applied, the Executive asked the student targeted questions regarding the policy window, one of the five streams of the MSF, which in turn deepened the student’s awareness of how the pandemic created an environment where decision-makers required expedited solutions to add hospital capacity. As a result of these discussions, the student came to understand the uniqueness of this policy window and gained appreciation that common barriers to policy development had been removed due to the pandemic environment, resulting in ability for policies to be made quickly. Further details outlining the application of the framework and the resulting benefit to the student’s learning are included in Table 1.

By applying a theoretical framework like the MSF to the practicum, the Executive also benefited. The application enabled the Executive to ground mentorship advise within a learning framework; to contextualize complex issues from different streams; to gain student advice on what they saw, given they were new to the organization and could offer fresh perspectives; and to personally reflect on the Executive’s work and how they might enhance the projects, given the framework and structured mentorship conversations create a natural reflection point on a specific project or policy.

## Conclusion

This case study demonstrates how the adoption of a theoretical policy framework, tied to both the student’s curriculum and the work environment, provided structure to align learnings against, and offered a common language for the Executive and student to discuss these learnings. Additionally, the framework helped to draw integration across various learning experiences throughout the practicum, and supported conversations regarding how ideas are generated and ultimately adopted into health policy. In order to replicate and further scale the learning benefits gained through introduction of the MSF to this practicum experience, universities offering such practicums, as well as students and Executive who are entering into practicum arrangements, should consider introducing a framework into their practicum foundation.

While this single case focused on the application of one theoretical framework, it is recognized other practicums and internships may be better experienced through application of other frameworks. As a result, future research in this area may include comparing the use of the MSF in multiple practicum environments, understanding the validity of different frameworks deployed within practicums, and comparing the value of practicums in environments where frameworks are used compared to those that do not deploy.
